# Histone deacetylase 7: a signalling hub controlling development, inflammation, metabolism and disease

**DOI:** 10.1111/febs.16437

**Published:** 2022-03-31

**Authors:** Yizhuo Wang, Rishika Abrol, Jeffrey Y. W. Mak, Kaustav Das Gupta, Divya Ramnath, Denuja Karunakaran, David P. Fairlie, Matthew J. Sweet

**Affiliations:** ^1^ 1974 Institute for Molecular Bioscience (IMB) The University of Queensland St. Lucia Australia; ^2^ 1974 IMB Centre for Inflammation and Disease Research The University of Queensland St. Lucia Australia; ^3^ 1974 Australian Infectious Diseases Research Centre The University of Queensland St. Lucia Australia

**Keywords:** class IIa HDAC, gene regulation, HDAC7, immunometabolism, macrophage

## Abstract

Histone deacetylases (HDACs) catalyse removal of acetyl groups from lysine residues on both histone and non‐histone proteins to control numerous cellular processes. Of the 11 zinc‐dependent classical HDACs, HDAC4, 5, 7 and 9 are class IIa HDAC enzymes that regulate cellular and developmental processes through both enzymatic and non‐enzymatic mechanisms. Over the last two decades, HDAC7 has been associated with key roles in numerous physiological and pathological processes. Molecular, cellular, *in vivo* and disease association studies have revealed that HDAC7 acts through multiple mechanisms to control biological processes in immune cells, osteoclasts, muscle, the endothelium and epithelium. This HDAC protein regulates gene expression, cell proliferation, cell differentiation and cell survival and consequently controls development, angiogenesis, immune functions, inflammation and metabolism. This review focuses on the cell biology of HDAC7, including the regulation of its cellular localisation and molecular mechanisms of action, as well as its associative and causal links with cancer and inflammatory, metabolic and fibrotic diseases. We also review the development status of small molecule inhibitors targeting HDAC7 and their potential for intervention in different disease contexts.

## Graphical abstract

Histone deacetylase 7 (HDAC7) is a lysine deacetylase and scaffolding protein that regulates numerous cellular and developmental processes via its multifaceted roles in signal transduction and gene regulation. This review summarises the many functions of HDAC7 in different cell types during homeostasis, our current understanding of associations between dysregulated HDAC7 expression and/or function and human disease, and the developmental status of small molecule HDAC7 inhibitors.

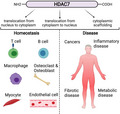

**Abbreviations**
AR: androgen receptor
CaMK: calcium/calmodulin‐dependent kinase
CBP/p300: cAMP‐response element‐binding protein‐binding protein
CFTR: cystic fibrosis transmembrane conductance regulator
CHDI: Cure Huntington's Disease Initiative
CRC: colorectal cancer
Crm1: chromosomal maintenance 1
CSCs: cancer stem cells
CtBP: C‐terminal‐binding protein 1
CYLD: cylindromatosis
DP: double positive
FOXO1: forkhead box protein O1
GBM: glioblastoma multiforme
GC: gastric cancer
*GENE*/*Gene*
: human gene name/mouse gene name
HAT: histone acetyltransferase
HCC: hepatocellular carcinoma
HD: Huntington's disease
HDAC: histone deacetylase
Hdac7‐s: Hdac7‐spliced
Hdac7‐u: Hdac7‐unspliced
HGF: hepatocyte growth factor
HIF‐1α: hypoxia‐inducible factor 1‐α
HUVEC: human umbilical vein endothelial cell
IBD: inflammatory bowel disease
iNKT: invariant natural killer T cell
MEF2: myocyte enhancer factor‐2
NSCLC: non‐small cell lung cancer
OA: osteoarthritis
PA: pancreatic adenocarcinomas
PD: Peyronie's disease
PKD: protein kinase D
PLZF: promyelocytic leukaemia zinc finger
PML: promyelocytic leukaemia protein
PROTEIN/Protein: human protein name/mouse protein name
PTM: post‐translational modification
SMRT: silencing mediator of retinoic acid and thyroid hormone receptor
SSc: systemic sclerosis
STAT: signal transducer and activator of transcription
T2D: type 2 diabetes
TCR: T cell receptor
TFMO: trifluoromethyloxadiazole group
TGF‐β1: transforming growth factor beta‐1
TLR: toll‐like receptor
VEGF: vascular endothelial growth factor
ZNF326: zinc‐finger protein‐326

## Introduction

### Regulated lysine acetylation and the histone deacetylase enzymes

Post‐translational modifications (PTMs), such as phosphorylation, ubiquitylation, glycosylation and acetylation, diversify protein functions, for example by altering protein structure, stability and activity. PTMs are critical in controlling cellular signalling pathways and their dysregulation is associated with many disorders [1, 2]. Reversible lysine acetylation regulates many biological processes, such as cell development [3], inflammation [4] and metabolism [5, 6]. This PTM is controlled by histone acetyltransferases (HATs) and histone deacetylases (HDACs), which respectively add and remove an acetyl moiety to and from the ɛ‐amino group of a lysine residue [7]. Acetylation neutralises the positive charge of a lysine residue in histones and is generally associated with chromatin decondensation, increased access to DNA by transcription factors and initiation of gene transcription [8, 9]. Conversely, histone deacetylation by HDACs is often linked to gene repression via chromatin condensation. However, this is an oversimplification of how histone acetylation influences chromatin architecture and gene expression. Moreover, proteomic‐based approaches on both tissues and cell lines have revealed thousands of non‐histone proteins that also undergo regulated lysine acetylation [10, 11]. Lysine acetylation occurs in numerous proteins, including metabolic enzymes, nuclear proteins and proteins involved in the cell cycle. Consequently, HATs and HDACs can both positively and negatively regulate gene expression and many other cell functions [12].

Human HDACs are grouped into four classes. Class I, II and IV HDACs, the so‐called classical HDACs (HDAC 1–11), all use zinc as a cofactor, whereas the class III HDACs (Sirtuins) act via an NAD^+^‐dependent mechanism [4]. The capacity of HDACs to deacetylate numerous proteins beyond histones (reviewed in Refs [12, 13]) enables them to participate in a plethora of cellular signalling pathways in different biological contexts. For example, class I and II HDACs control a number of cardiovascular functions [14, 15], while specific class II HDACs regulate skeletogenesis [3]. Class I HDACs are primarily localised to the nucleus, where they control gene expression [16]. HDAC11 is the sole class IV member. It is expressed by multiple tissues and cells and regulates differentiation, migration and inflammatory responses in immune cells [17]. The class II HDACs share sequence similarity to yeast Hda1 and are subdivided into the class IIa HDACs (HDAC4, 5, 7 and 9) and the class IIb HDACs (HDAC6, HDAC10) [18]. The roles of class IIa HDACs in physiological and pathological processes have previously been reviewed [19, 20]. This review focuses on the biology and disease associations of HDAC7, a specific class IIa HDAC with important roles in development, immune responses, inflammation, metabolism and the vascular system.

### Class IIa HDACs and HDAC7

Class IIa HDACs have a highly conserved C terminus that contains a deacetylase domain and a nuclear export signal. The N terminus, which is less conserved between members of this sub‐family [18], contains a nuclear localisation signal and a domain that interacts with a broad range of cellular proteins [21]. As with other classical HDACs, class IIa HDACs have a tubular substrate‐binding pocket with the zinc cofactor at the end of this tunnel [22]. A tyrosine and two histidine residues in the active site are particularly important for enzymatic activity of most classical HDACs [9, 23]. However, the tyrosine is replaced by a histidine in the class IIa HDACs, considerably reducing enzymatic activity for lysine deacetylation on specific peptide substrates [24]. Currently, there is controversy over whether class IIa HDACs actually deacetylate histones at all in cells [25]. They do efficiently process artificially activated (trifluoroacetyl)lysine‐containing substrates *in vitro*, but early studies pointed to class IIa HDACs not being deacetylating enzymes in the nucleus. More recent work has shown that specific stimuli do induce class IIa HDAC enzyme activity in the cytosol of cells [26], and several other studies support the idea that these enzymes can deacetylate lysine residues of non‐histone proteins. For example, HDAC4 and HDAC5 reportedly deacetylate the transcription factors signal transducer and activator of transcription 1 (STAT1) [27], forkhead box protein O1 (FOXO1) and FOXO3 [28], HDAC7 deacetylates the glycolytic enzyme PKM2 [29] and HDAC9 deacetylates the transcription factor USF‐1 [30]. An interaction with the class I HDAC, HDAC3, could potentially contribute to some of these enzyme‐dependent functions [31], and it is also possible that class IIa HDACs carry out alternative PTMs to lysine deacetylation. Nonetheless, based on the mounting evidence above, it seems likely that class IIa HDACs can indeed function as active lysine deacetylases in at least some circumstances.

**Fig. 1:**
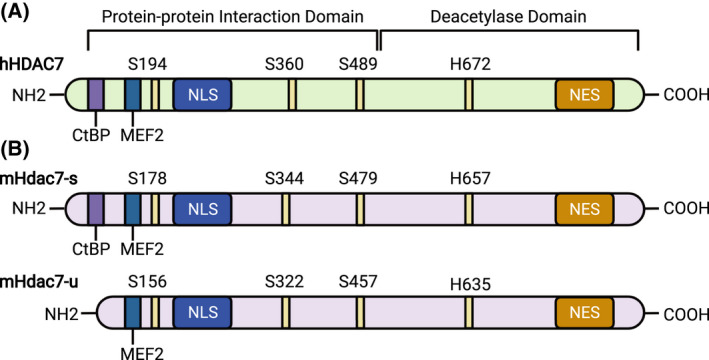
Schematic diagram of the human and mouse HDAC7 proteins. Schematic diagrams of the human HDAC7 protein (hHDAC7) (A) and the mouse Hdac7 protein (mHdac7) isoforms that are generated by alternative splicing (B). Hdac7‐spliced (Hdac7‐s) is a full‐length protein generated after excision of a 57 bp intron region. This intron is retained in the Hdac7‐unspliced (Hdac7‐u) isoform, resulting in the presence of premature start codons and use of an alternate downstream translation start site. Consequently, Hdac7‐u lacks the first 22 N‐terminal amino acids (and a binding site for the transcriptional repressor CtBP) that are present in Hdac7‐s. Serine (S) residues that regulate HDAC7 nuclear/cytoplasmic shuttling, as well as a histidine (H) residue that is essential for enzymatic activity, are indicated in yellow and are numbered in the context of the indicated protein (e.g. S178 in mHdac7‐s corresponds to S156 in mHdac7‐u). Proteins used for amino acid numbering are NM_001098416.4 (hHDAC7), NP_062518.2 (mHdac7‐s) and NP_001191207.1 (mHdac7‐u). MEF2, binding site for members of the MEF2 transcription factor family; CtBP, binding site for C‐terminal binding protein; NLS, nuclear localisation signal; NES, nuclear export signal.

The human *HDAC7* gene is located on chromosome 12q31 [32] and encodes a polypeptide of 912 amino acids. Human HDAC7 shares 95% similarity at the amino acid level with murine Hdac7 [33]. Figure 1 highlights specific domains and amino acids in HDAC7 that are important for its biological functions. The mouse Hdac7 protein was first described as an HDAC that is recruited to the silencing mediator of retinoic acid and thyroid hormone receptor (SMRT), otherwise known as nuclear receptor co‐repressor 2, Ncor2, in the nucleus [34]. This direct interaction enables the formation of a protein complex containing another class IIa member Hdac5 and the co‐repressor mSin3A, with this multimeric complex acting as a repressor of gene expression. The human HDAC7 protein was initially reported to be present in both nucleus and cytoplasm of human muscle, neuroblastoma and mouse embryo cell lines when overexpressed, with the nuclear form having a selective role in repressing gene expression via an interaction with corepressors SMRT and N‐CoR [33]. Its enzyme activity in the nucleus was shown to rely on binding to HDAC3, whereas cytoplasmic HDAC7 was considered enzymatically inactive as it did not bind to this class I HDAC. The original crystal structure of the catalytic domain of human HDAC7 revealed an enlarged active site pocket and a zinc‐binding structural motif away from its active site, with this being conserved in other class IIa HDACs [22]. Despite this conservation, HDAC7 appears to have several unique properties. For example, HDAC7 has been reported to deacetylate the transcription factor STAT3 [35] and the proinflammatory metabolic enzyme PKM2 [29] and appears to be the sole class IIa HDAC enzyme that is activated in macrophages responding to inflammatory stimuli [26]. In addition, HDAC7 can also promote SUMOylation of target proteins. Overexpression of HDAC7 in HEK293 cells resulted in SUMOylation of MEF2D, a member of the myocyte enhancer factor‐2 (MEF2) family of transcription factors, thus inhibiting its function as a transcriptional activator [36]. Similarly, in human endothelial cells, HDAC7 colocalised with promyelocytic leukaemia protein (PML) within nuclear bodies, promoting its SUMOylation via a SUMO E3 ligase‐like activity [37]. This modification was independent of the deacetylase activity of HDAC7, relying instead on direct scaffolding with the SUMO E2 enzyme Ubc9. Given the diverse functions of PML nuclear bodies in different cellular processes [38], this SUMOylation‐promoting activity of HDAC7 may be important. The capacity of HDAC7 to interact with many different proteins (summarised in Table 1) likely contributes to its unique roles in several biological processes.

**Table 1:** Interaction partners of HDAC7 and gene targets. Co‐IP, co‐immunoprecipitation; ChIP, chromatin immunoprecipitation; PLA, proximity ligation assay; RIME, rapid immunoprecipitation mass spectrometry of endogenous protein.

Cellular process	Effect on target gene	Cell type	Interaction partner(s)	Evidence	Reference(s)
T cells					
TCR‐mediated apoptosis	*Nur77* repression	DO11.10 T cell hybridomas, HEK293T	MEF2D, PKD	Reporter assays, Co‐IP, GST pull down	[44, 45, 49, 58]
Disruption of regulatory T cell function via IL‐2	N/A	HEK293T, Jurkat E6.1 T cells, primary CD4^+^CD25^+^ T cells	FOXP3	Co‐IP, reporter assays	[183]
Development of iNKT cells	Repression of PLZF transcriptional activity	Mouse thymocytes, HEK293T	PLZF	Co‐IP, reporter assays	[72]
B cells					
B cell apoptosis	*MYC* repression	Namalwa	MEF2C, HDAC3, SMRT	Co‐IP	[46]
Suppression of pre‐B cell trans‐differentiation into macrophages	*Mac‐1*, *Itgam*, *Fcgr1*, *Ccl3* repression	HAFTL	MEF2C	Co‐IP, ChIP	[74]
Macrophages					
TLR‐inducible inflammatory responses	Promotion of Hif‐1α‐dependent gene expression	HEK293T, BMM, RAW 264.7	Hif‐1α, Pkm2, CtBP	Co‐IP, PLA, AlphaLISA, RIME	[29, 63]
Endothelial cells					
Suppression of endothelial cell proliferation	Downregulation of β‐catenin target genes	HUVEC	β‐catenin, 14‐3‐3 ε, ζ and η	Co‐IP	[54]
Maintenance of vascular integrity	Repression of MEF2C‐inducible matrix metalloproteinase 10 (*MMP10*)	HAEC, COS‐1	N/A	ChIP, reporter assays	[43]
Suppression of endothelial cell proliferation and migration	Repression of MEF2‐dependent *NUR77* expression and repression of *RCAN2*	HAEC, HUVEC	14‐3‐3	Co‐IP	[85]
PML SUMOylation to promote nuclear body formation	N/A	HUVEC	SUMO E2 enzyme Ubc9	Co‐IP	[37]
Promotion of angiogenesis	Repression of STAT3‐dependent *AKAP12* and *ICAM1* expression	HUVEC	N/A	ChIP	[87]
Other cell types					
MEF2D SUMOylation	NA	HEK293	MEF2D	Co‐IP	[36]
MEF2 activity repression in myogenesis	NA	Cos‐7	MEF2C, CtBP	Co‐IP, GST pull down	[42]
Transcriptional regulation of HIF‐1α during hypoxia	HIF‐1α‐dependent transcriptional activation of *VEGF* and *Glut‐1*	HEK293	HIF‐1α, CBP/p300	Co‐IP, yeast two‐hybrid assay, immunofluorescence	[56]
Androgen receptor (AR)‐mediated gene repression	Prostate‐specific antigen repression	HeLa, LNCaP	AR	Co‐IP, immunofluorescence and reporter assays	[57]
Repression of gastrointestinal histidine decarboxylase promoter activity	KLF4‐mediated gene repression	AGS	KLF4, TIP60	Co‐IP, reporter assays, ChIP	[184]
Gene regulation	Repression of STAT3‐regulated gene expression	HEK293, HepG2	TIP60	Co‐IP, reporter assays	[185]
Inhibition of cell density‐dependent inflammatory responses	Repression of cyclooxygenase 2 (*COX‐2*) expression	HEK293T, H358	YAP/TAZ, TEAD	Co‐IP, ChIP	[186]
Gene regulation	Transcriptional repression of *RPRM*	MCF7, HEK293T, Ly2	Estrogen receptor α, FoxA1	Co‐IP, ChIP	[187]
Lung tumour cell proliferation and apoptosis	Repression of STAT3‐mediated gene transcription	H1299, mouse lung tumours, HEK293T, A549	STAT3	Co‐IP, GST pull down	[35]
Repression of MEF2 activity	N/A	HEK293, NIH3T3	MEF2A, C, D, 14‐3‐3 ε	Co‐IP, reporter assays, GST pull down	[48]
Inhibition of osteoclast differentiation	*Nfat‐c1*, *Ctsk*, *DC‐STAMP* transcriptional repression	RAW 264.7 c4, primary murine osteoclasts	Mitf	Co‐IP, reporter assays	[77]
Inhibition of osteoblast maturation	Repression of osteoblast marker genes	ROS 17/2.8, C2C12	Runx2	Co‐IP, reporter assays	[80]
Inhibition of chondrocyte proliferation	Repression of β‐catenin target genes	ATDC5	β‐catenin	Co‐IP	[83]

## HDAC7 functions are dependent on cellular localisation

### Nuclear to cytoplasmic shuttling

**Fig. 2:**
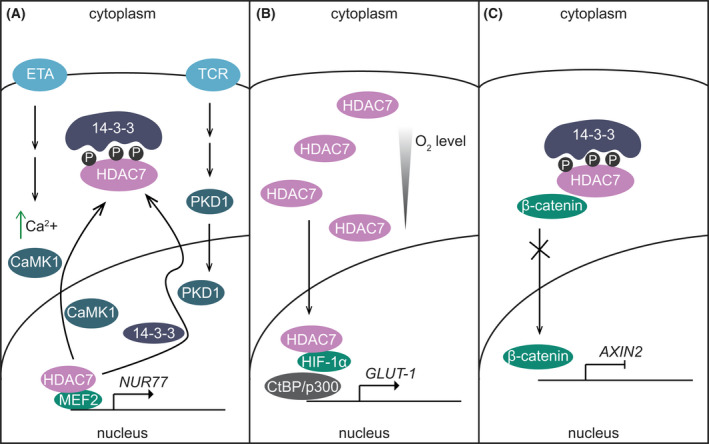
Subcellular localisation of HDAC7 results in distinct biological functions. (A) Nuclear export of HDAC7 enables inducible gene expression, via its action as a transcriptional derepressor. In response to increased Ca^2+^ concentrations or upon TCR activation, nuclear HDAC7 is phosphorylated by CaMK1 or PKD1, respectively, resulting in its nuclear export. Cytoplasmic HDAC7 binds to 14‐3‐3 at phosphorylated residues, with the nuclear export of HDAC7 enabling derepression of MEF2‐regulated genes such as *NR4A1* (*NUR77*) in T cells and other cell types. (B) Nuclear import of HDAC7 results in inducible gene expression, via its action as a transcriptional activator. Under hypoxia, HDAC7 shuttles into the nucleus and binds to HIF‐1α and CBP/p300, initiating expression of HIF‐1α target genes such as *SLC2A1* (*GLUT1*) in HEK293 cells. (C) The cytoplasmic HDAC7/14‐3‐3 complex retains β‐catenin in the cytoplasm, preventing its translocation into the nucleus, thus limiting expression of β‐catenin‐dependent genes such as *AXIN2*. Cytoplasmic functions of HDAC7 have been observed in multiple cell types, for example HUVEC and macrophages. CaMK1, calcium/calmodulin‐dependent kinase 1; ETA, endothelin receptor A; HIF‐1α, hypoxia‐inducible factor 1‐α; MEF2, myocyte enhancer factor‐2; PKD1, protein kinase D1; TCR, T cell receptor.

Due to the presence of both nuclear localisation and nuclear export signals, HDAC7 executes different cellular functions depending on the cell type and environmental cues received (Fig. 2). Within the nucleus, HDAC7 generally inhibits gene expression by forming repressor complexes with transcription factors and co‐regulators [18]. The most extensively characterised interaction partners of HDAC7 in this context are members of the MEF2 family of transcription factors, with various class IIa HDACs being critical regulators of MEF2‐dependent gene transcription [39]. MEF2 family members (MEF2A, MEF2B, MEF2C, MEF2D) activate or repress genes responsible for cell division, differentiation or death in numerous calcium‐dependent signalling pathways (reviewed in Refs [40, 41]). The N‐terminal region of HDAC7 has a conserved binding motif for MEF2C, with this enabling the repression of various MEF2‐dependent genes involved in many biological processes such as muscle differentiation [42], vascular integrity [43], thymocyte development [44, 45] and oncogenesis [46]. Repression of MEF2‐dependent gene expression involves the recruitment of co‐repressors such as C‐terminal‐binding protein 1 (CtBP) [42], HDAC3 [33, 46] or SMRT [46]. Specific signalling events, for example activation through the T cell receptor (TCR) [44], promote nuclear export of HDAC7 to enable MEF2‐dependent gene expression. This phenomenon is often referred to as transcriptional derepression.

Nuclear/cytoplasmic shuttling of class IIa HDACs is regulated by both calcium‐dependent and calcium‐independent mechanisms [47]. Members of the calcium/calmodulin‐dependent kinase (CaMK) [48] and protein kinase D (PKD) [49] families are able to phosphorylate HDAC7 on specific serine residues, thus enabling its nuclear export. For example, CaMK‐mediated serine phosphorylation of Hdac7 enables an interaction with the 14‐3‐3ε chaperone protein and its chromosomal maintenance 1 (Crm1)‐dependent nuclear export, thus permitting MEF‐inducible gene expression through transcriptional derepression [48]. Based on a previous study showing that the potent vasoconstrictor endothelin‐1 triggered nuclear export of HDAC7 from COS‐7 cells [50], it was proposed that endothelin‐1 may initiate this response via CaMK [48]. However, most studies on nuclear‐cytoplasmic shuttling of HDAC7 have focused on CaMK‐independent mechanisms. For example, PKD1 phosphorylates HDAC7 via a calcium‐independent mechanism leading to its translocation to the cytoplasm in T cells [44, 49]. Other protein kinases that were reported to phosphorylate HDAC7 include PKD2 in human gastric cancer cells [51], PKD1 and PKD3 in B cells [52] and liver kinase B1‐dependent AMP‐activated protein kinase in liver [28]. Furthermore, ectopic expression experiments in HeLa cells have revealed that CRM1‐mediated nuclear export of HDAC7 can also occur independently of serine phosphorylation [53]. This study showed that CAMK did not phosphorylate a S178A/S344A/S479A HDAC7 mutant, but this mutant was still exported by CRM1 to the cytoplasm. Additionally, a S178E/S344E/S479E phosphorylation mimic was unable to associate with the 14‐3‐3ε scaffold protein but was still exported to the cytoplasm via CRM1. This suggests that the association with 14‐3‐3ε is not necessarily essential for CRM1‐mediated HDAC7 export and/or cytoplasmic retention, although it is noted that these studies employed artificial overexpression systems. What is clear is that, depending on the cell type and environmental stimulus, signal‐induced nuclear export of HDAC7 can occur via multiple mechanisms.

### Cytoplasmic functions

In some cells, HDAC7 constitutively localises to the cytoplasm, where it executes specific functions. In this context, cytoplasmic HDAC7 plays important roles in regulating cell development and inflammation by indirectly repressing or inducing gene expression. Prior to cellular activation, HDAC7 scaffolds β‐catenin, a promoter of proliferation, in the cytoplasm of human endothelial cells [54]. Cytoplasmic retention of β‐catenin inhibits its nuclear translocation, thus limiting its expression of target genes and cellular proliferation. In rodent cardiomyocytes, cardiac stress resulted in salt‐induced kinase I‐mediated phosphorylation of cytoplasmic Hdac7, thus preventing its degradation and enabling it to contribute to cardiac pathology through indirect activation of Myc‐inducible stress‐related genes [55]. Several studies have also demonstrated roles for cytoplasmic Hdac7 in both innate and adaptive immune cells (see ahead).

### Cytoplasmic to nuclear shuttling

Whereas many early studies focused on nuclear export of HDAC7 and transcriptional derepression, it has also been revealed that this lysine deacetylase can translocate to the nucleus from the cytoplasm to activate gene expression. One of the first studies to identify such activity investigated cellular responses to hypoxia in HEK293 cells [56]. Following induction of hypoxia, hypoxia‐inducible factor 1‐α (HIF‐1α) bound to HDAC7, translocating to the nucleus and forming a complex with transcriptional co‐activator cAMP‐response element‐binding protein‐binding protein (CBP)/p300 that permitted inducible gene expression. HDAC7 has also been shown to translocate to the nucleus in response to other cell signals, for example steroid hormones. An androgen receptor (AR) agonist triggered HDAC7 translocation from the cytoplasm into the nucleus in HeLa cells, with HDAC7 forming a complex with AR to repress its transcriptional activator function [57]. This inhibitory effect was independent of AR deacetylation but was related, in part, to deacetylation of the co‐activator CBP. Furthermore, in PMA‐treated or TCR‐activated mouse thymocytes, the myosin phosphatases PP1β and MYPT1 dephosphorylated Hdac7 after the initial wave of signalling, resulting in its relocation back to the nucleus. This restored the repression of *Nur77*, thus limiting thymocyte apoptosis [58].

### Mitochondrial functions

In addition to its nuclear and cytoplasmic functions, one study reported that HDAC7 localises to the inner mitochondrial membrane of prostate epithelial cells, due to the presence of a mitochondrial targeting sequence in its N terminus [59]. After the initiation of apoptosis, it was released into the cytoplasm where it was postulated to be involved in apoptosis. However, no further reports have linked HDAC7 to mitochondrial biology, so the significance of this finding is unclear at this stage.

## HDAC7 functions in different cell types

**Fig. 3:**
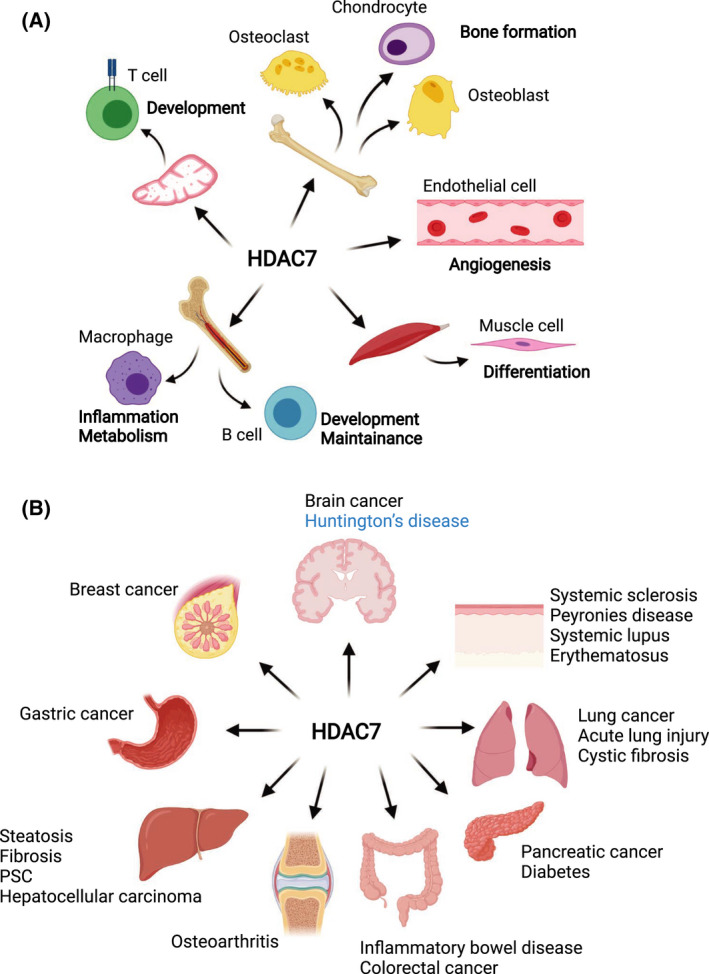
Roles of HDAC7 in physiological and pathophysiological processes. (A) HDAC7 regulates the functions of many cell types in response to a range of differentiation and/or activation signals (see Table 1 for specific transcription factors involved). This class IIa HDAC regulates cell fate choices during both T cell and B cell development, as well as vascular formation and angiogenesis during embryonic development. It also controls the functions and stress responses of cardiomyocytes, as well as metabolic and inflammatory responses in innate immune cells. (B) Because of its diverse physiological roles, dysregulated expression and/or function of HDAC7 has been implicated in numerous pathologies. These include cancer (both solid tumours and leukaemias), as well as inflammatory and metabolic diseases that affect the liver, digestive systems, lung and joints. In contrast, HDAC7 may have a protective role in limiting neurodegenerative disease progression in Huntington's disease (labelled in blue). PSC, primary sclerosing cholangitis.

HDAC7 regulates the functions of numerous cell types, thereby influencing a diverse range of physiological and pathophysiological processes (Fig. 3; Table 2). For example, extensive literature has documented involvement of HDAC7 in both T cell and B cell development [60, 61], as well as in lymphocyte activation [62]. More recent studies have revealed key roles for HDAC7 in inflammation, through its functions in Toll‐like receptor (TLR)‐activated macrophages [26, 29, 63]. In non‐immune cells, such as endothelial cells and muscle cells, HDAC7 has several important roles. It is required for blood vessel development and vascular integrity during embryonic development [43] and also contributes to cardiac stress responses through its functions in cardiomyocytes [55]. Below we provide an overview of the functions of HDAC7 in different cell types, focusing on how subcellular localisation of this class IIa HDAC influences function.

**Table 2:** Biological functions of HDAC7.

Signal	Cell type	Species	Effects/functions	Reference(s)
T cell receptor activation	DP thymocyte	Mouse	Promotes positive/negative selection	[60]
	CD8^+^ T cell		Promotes Ifnγ production	[67]
	CD4^+^ T cell		Promotes *Nurr77* and *Irf4* expression	[68]
	iNKT cell		Promotes thymocyte to iNKT cell development	[72]
B cell receptor activation	B cell	Mouse	Cell development	[61, 74]
Tlr4 activation	Macrophage	Mouse	Promotes proinflammatory cytokine Il‐1β and Ccl2 secretion, TLR‐inducible glycolysis	[26, 29]
Growth factor‐induced differentiation	Osteoclasts, bone marrow	Mouse	Inhibits osteoclast differentiation and precursor proliferation	[77, 78, 79]
Osteoblast differentiation	Myoblast, fibroblast	Human Mouse	Inhibits osteoblast differentiation	[80, 81, 82]
Growth factor‐induced proliferation	Chondrocytes	Mouse	Inhibits chondrocyte proliferation	[83]
VEGF signalling	Endothelial cell	Mouse Human	Promotes cell proliferation and migration	[43, 84, 85, 86, 88]
		Human	Maintains vascular integrity	[54, 87, 88]
Smooth muscle cell differentiation	Muscle cell	Mouse	Promotes myoblast migration and myocyte differentiation	[90, 91, 92]
Cardiac hypertrophy	Cardiomyocyte	Human Mouse	Contributes to pathologic cardiac remodelling	[55]

### T lymphocytes

Some of the first studies to characterise HDAC7 functions in lymphocytes focused on T cell development. Following migration of committed lymphoid progenitors to the thymus, these progenitors first proliferate and acquire a CD4/CD8 double‐positive (DP) phenotype and undergo gene rearrangement to express a functional TCR. The intensity of TCR signalling then decides cell fate, with successive rounds of positive and negative selection permitting survival of cells with an intermediate level of TCR signalling. These cells then downregulate one of the TCR coreceptors to become either CD4^+^ helper or CD8^+^ cytotoxic T cells [64]. The majority of developing thymocytes die by apoptosis during positive and negative selection, thus preventing the generation of both non‐functional and autoreactive T cells [65]. The control of thymocyte survival and cell death is therefore critical for normal immune function. In the resting state, DP mouse thymocytes express high levels of nuclear Hdac7 [45], enabling it to repress the expression of T lymphocyte differentiation and pro‐apoptotic genes such as *Nur77* via Mef2d [45]. Following TCR activation, phosphorylation of Hdac7 on multiple serine residues by Pkd1 results in the disruption of its interaction with Mef2d and its nuclear export [44, 49]. This enables inducible expression of *Nur77* and initiation of cellular apoptosis. Consistent with this, a phosphorylation‐defective mutant of Hdac7 was impaired in its ability to activate Erk1/2 and p38 downstream of TCR signalling, resulting in defective negative selection [66]. Hdac7 also contributes, to a lesser extent, to positive selection of thymocytes. Blocking nuclear export of Hdac7 modestly impaired positive selection [66], with studies on *Hdac7*‐deficient thymocytes also supporting a role for this class IIa HDAC enzyme in positive selection [62]. In addition, global transcriptional profiling in a T cell line revealed that many HDAC7 target genes are associated with these processes [60]. Collectively, these studies have revealed that HDAC7 has essential roles in regulating cell fate choices during thymocyte development.

HDAC7 also regulates the functions of mature T cells. In contrast to its localisation in immature thymocytes, HDAC7 accumulates in the cytoplasm of specific populations of mature T cells. This cytoplasmic localisation of HDAC7 occurs through constitutive serine phosphorylation, enabling transcriptional derepression of genes crucial for mature T cell functions. In CD8^+^ T cells, Hdac7 was constitutively phosphorylated on serine 178, independent of any TCR activation signal [67]. Cytoplasmic localisation of Hdac7 was essential for functional responses of CD8^+^ T cells, for example TCR‐mediated Ifnγ production. In CD4^+^ T cells, HDAC7 was also serine phosphorylated, with this being dependent on the transmembrane adaptor protein, Linker for Activation of T cells [68]. Tonic signalling through this adaptor leads to gamma phospholipase C‐mediated phosphorylation of Hdac7 and its cytoplasmic accumulation, enabling expression of the pro‐apoptotic gene *Nur77*, as well as *Irf4*. Conversely, nuclear HDAC7 suppresses gene expression and its associated CD4^+^ T cell functions [68]. Taken together, these studies highlight roles for HDAC7 in both T cell development and in the functions of mature T cells.

During T cell development, thymocytes can follow an alternative developmental pathway to become invariant natural killer T cell (iNKT) cells. Rather than recognising peptides presented by MHC‐like conventional T cells, iNKT cells respond to lipids presented by CD1d and can be rapidly activated during immune activation [69, 70]. Unlike conventional T cells that retain their phenotype while leaving the thymus, these cells are comparatively larger and possess an antigen‐experienced phenotype that can in part be attributed to expression of the transcription factor promyelocytic leukaemia zinc finger (PLZF) [71]. As described above, HDAC7 is highly expressed in DP thymocytes, with its functions being influenced by its phosphorylation status. In phosphorylation‐defective Hdac7 mutant mice, where Hdac7 localised in the nucleus of developing thymocytes, iNKT cell survival was compromised and mice developed autoimmunity [72]. These mice also developed phenotypes resembling human inflammatory conditions, for example inflammatory bowel disease. Following TCR stimulation, nuclear Hdac7 in the phosphorylation‐defective mutant mice acts as a transcriptional repressor of PLZF, which diverts the iNKT cell fate to rare naïve T cells. This suggests that phosphorylation of HDAC7 plays a crucial role in determining cell fate, particularly iNKT cell development and survival, with this being important in limiting autoimmunity and inflammatory disease.

### B lymphocytes

B cell differentiation is a tightly regulated process associated with epigenetic modifications and alterations in transcription factor expression and/or function. As with T cells, MEF2 family members have important roles in regulating B cell development and differentiation [73]. HDAC7 is highly expressed in lymphoid progenitors, including pre‐B cells, where it binds to MEF2C and represses the expression of myeloid genes such as *CD11b* [74]. This function provides a mechanism for maintaining B cell identify during development. Deletion of *Hdac7* in lymphoid progenitors has also been associated with a block in early B cell development, coupled with reduced cell viability [61].

### Innate immune cells

Emerging evidence has documented distinct roles for HDAC7 in both myeloid development and function. As noted above, Hdac7 negatively regulates the expression of myeloid genes during mouse B cell development [74]. That study also showed that low levels of Hdac7 were required for the trans‐differentiation of B cells to macrophages. Indeed, ectopic expression of Hdac7 interfered with macrophage‐specific lineage acquisition. Consistent with this study, we previously found that RAW 264.7 mouse macrophage‐like cells express low levels of *Hdac7* [63]. However, despite its inhibitory effect on myeloid cell development, at least in a B cell to macrophage transdifferentiation model, Hdac7 does have important roles in regulating the functions of mature macrophages. It was expressed at elevated levels by inflammatory macrophages by comparison to other macrophage populations [63]. This study also showed that ectopic expression in RAW264.7 cells of an Hdac7 isoform lacking 22 amino acids at the N terminus (Hdac7‐unspliced, Hdac7‐u), which is unable to bind to the transcriptional repressor Ctbp, enhanced the TLR‐inducible expression of a subset of inflammatory genes. Recent studies have also revealed that HDAC7 localises to the cytoplasm of both human and murine macrophages [26], with TLR‐mediated activation of cytoplasmic Hdac7 in murine macrophages enabling inducible expression of *Il1b* that encodes the pro‐inflammatory cytokine IL‐1β [29]. The mechanism involves Hdac7‐mediated deacetylation of the glycolytic enzyme Pkm2, enabling activation of the transcription factor Hif‐1α and initiation of inflammatory gene expression. Hdac7 also promotes TLR‐inducible glycolysis [26, 29], a metabolic response increasingly linked to inflammation [75]. HDAC7 is thus emerging as an important initiator of innate immune responses.

### Osteoclasts and osteoblasts

Bone homeostasis is maintained by bone resorption and bone formation, processes controlled by osteoclasts and osteoblasts, respectively [76]. HDAC7 promotes bone formation by inhibiting osteoclast differentiation, as well as promoting osteoblast development and endochondral ossification in response to various growth factors and cytokines. In osteoclasts, Hdac7 directly suppressed the transcription factors Mitf, β‐catenin and NFATc1 [77, 78, 79]. Such effects enabled Hdac7 to suppress both precursor proliferation and osteoclast differentiation. Therefore, Hdac7 promotes bone formation by inhibiting two stages of osteoclastogenesis. In osteoblasts, nuclear HDAC7 acts as a transcriptional repressor to inhibit cell differentiation. Signal‐induced phosphorylation of Hdac7 resulted in its cytoplasmic accumulation in these cells, enabling activation of the pro‐osteogenic Runx2 transcription factor that supports osteoblast development and maturation [80, 81, 82]. Another study also showed that Hdac7 suppressed β‐catenin functions in chondrocytes, thus limiting chondrocyte proliferation during endochondral ossification [83]. Hence, HDAC7 acts by multiple mechanisms in multiple cell types to regulate bone homeostasis.

### Endothelial cells

During the formation of new blood vessels through angiogenesis, activated endothelial cells proliferate and migrate into the extracellular matrix to form new tubules. Early studies on *Hdac7*‐deleted mice revealed its essential role in blood vessel development and maintenance of vascular integrity [43]. Endothelial cell‐specific deletion of *Hdac7* led to vascular defects such as blood vessel enlargement and cell–cell adhesion failure [43], identifying its intrinsic role in vascular development. Genetic loss‐of‐function studies in HUVECs and umbilical cord blood mononuclear cell‐derived endothelial progenitor cells [84] also showed that HDAC7 promotes migration and tube‐forming capacity. Activation of endothelial cells with vascular endothelial growth factor (VEGF) resulted in PKD1‐mediated phosphorylation of HDAC7, its nuclear export and inducible expression of VEGF‐responsive genes including *NUR77, RCAN2* [85] and angiogenic matrix metalloproteinases [86], thus enabling cell proliferation and migration. HDAC7 also supported angiogenesis by limiting the expression of the angiogenesis suppressor gene *AKAP12* in the nucleus [87]. Similarly, HDAC7‐mediated suppression of *PDGF‐B* and its receptor *PDGFR‐β* enabled optimal endothelial cell migration, again supporting angiogenesis [88].

In contrast to the pro‐angiogenic roles for nuclear HDAC7 highlighted above, cytoplasmic HDAC7 can also have anti‐angiogenic functions [54]. Ectopic expression of HDAC7 in HUVECs sequestered β‐catenin in the cytoplasm, blocked G1/S phase transition and inhibited cell proliferation, whereas knockdown of HDAC7 by shRNA enhanced the nuclear translocation of β‐catenin and endothelial cell growth. This study also showed that VEGF treatment promoted HDAC7 degradation, enabling translocation of β‐catenin to the nucleus to induce gene expression and to support cell growth, further confirming an anti‐proliferative function for HDAC7 in this context. Such data again highlight that the subcellular localisation of HDAC7 is critical in dictating its function.

### Muscle cells

Myogenesis is a complex process that involves the differentiation of stem cells to specialised muscle cells [89]. HDAC7 plays a crucial role in myogenesis by regulating the migration and differentiation of myoblasts [90]. Nuclear Hdac7 limited myoblast differentiation by repressing Mef2c‐mediated transcription [42]. Hdac7 was present in both the nucleus and cytoplasm of myocytes but was exclusively cytoplasmic after differentiation. Preventing nuclear export of Hdac7 disrupted myogenesis, likely through repression of MEF2 transcription factor function [90]. This highlights another example of the importance of nuclear export of HDAC7 in cell differentiation pathways. Subsequent studies further demonstrated that nuclear versus cytoplasmic targeting of Hdac7 was crucial for controlling embryonic stem cell differentiation into smooth muscle cells [91, 92]. *Hdac7* mRNA undergoes differential splicing to generate both an unspliced (Hdac7‐u) and a spliced isoform (Hdac7‐s), with these isoforms binding and regulating different transcription factors in different cellular compartments [91]. In the early stage of stem cell differentiation, Hdac7‐u supported proteasomal degradation of Mef2c in the cytoplasm, leading to gene repression and differentiation towards non‐muscle cells. In response to specific stimuli such as platelet‐derived growth factor, Hdac7‐s bound the transcription factor SRF in the nucleus, leading to gene expression and differentiation towards muscle cells. Thus, these two isoforms of Hdac7 appear to differentially regulate myogenesis.

Hypertension and other pathological factors can cause cardiac stress [93], resulting in abnormal gene expression and stress‐dependent pathological remodelling of cardiac tissue. In contrast to the other class IIa HDACs that repress MEF2‐dependent transcription during such events, HDAC7 has a distinct role in promoting hypertrophic signalling in cardiomyocytes [55]. Although Hdac7 was exclusively cytoplasmic, it indirectly induced expression of the c‐Myc transcription factor to drive the cardiomyocyte stress response, as well as pathological cardiac remodelling and induction of genes linked to heart failure. The stability of Hdac7 was dependent on its phosphorylation by the serine/threonine kinase salt‐inducible kinase 1. HDAC7 thus has a unique role, distinct from other class IIa HDACs, in driving cardiac pathology. Further studies on HDAC7 in this context may reveal opportunities for limiting pathological remodelling of the heart.

## HDAC7 association with human disease

Members of the HDAC family that regulate inflammation [4, 94] have been linked to pathology in a number of animal models of inflammatory diseases and cancer [3, 95] and have been pursued as targets in human disease. Below we review literature that specifically links HDAC7 to different conditions through genetic association and/or animal model studies (summarised in Table 3).

**Table 3:** Links between HDAC7 and disease.

Disease	Species/Model	Phenotype/evidence	Mechanism	Reference(s)
Cancer				
Colorectal cancer	Human/patient tissues and cell line	*HDAC7* was overexpressed in colorectal cancer and associated with cancer progression	HDAC7 promotes CRC cell line proliferation and invasion	[96, 104]
	Mouse/adenovirus‐mediated transfection	Hdac7 overexpression in mice promoted tumour growth	N/A	[104]
Pancreatic cancer	Human/patient tissues and cell line	Higher *HDAC7* in cancerous tissues associated with reduced survival, distant metastasis, poor prognosis	Silencing *HDAC7* by shRNA in cell line inhibits tumour cell growth	[99, 105]
Hepatocellular carcinoma	Human/patient tissues and cell line	*HDAC7* was upregulated in HCC cell lines and liver tumours, correlates with poor prognosis	Pharmaceutically inhibiting HDACs impedes the growth and migration of HCC cell lines	[106]
	Mouse/*Trim24^−/−^* mice tissues and cell line	*Hdac7* was upregulated in HCC cell lines and liver tumours		
Glioblastoma multiforme	Human/patient tissues and cell line	Upregulated *HDAC7* in patients was linked to poor survival	HDAC7 suppresses JAK1, AKAP12, STAT3, and deacetylates β‐catenin in the Wnt oncogenic pathway. *HDAC7* silencing induces anti‐angiogenic genes and inhibits pro‐angiogenic genes	[100, 114]
	Chicken and mouse/chorioallantoic membrane assay model	*Hdac7* silencing inhibited angiogenesis and tumour growth	Hdac7 promotes tumour growth and proliferation	[114]
Lung cancer	Human/patient tissues and cell line	*HDAC7* was dysregulated in tumour tissues. Higher *HDAC7* correlated with poor patient survival. *HDAC7* knockdown enhanced apoptosis	HDAC7 deactivates STAT3‐mediated tumour suppressive function and promotes neovascularisation	[35, 122, 188]
	Mouse/xenograft models of NSCLC and Ad‐Cre‐ or lenti‐Cre‐mediated *LSL‐K‐Ras^G12D^* mouse	*Hdac7* silencing reduced tumour number and cell proliferation, but increased apoptosis	Genetic targeting of *Hdac7* increases Stat3 phosphorylation in lung tumours and reduces tumour growth	[35, 122]
Gastric cancer	Human/patient tissues and cell line	*HDAC7* was dysregulated in cancerous tissues and correlated with Ki‐67 expression, distant metastasis and poor prognosis	HDAC7 may promote cancer cell proliferation by activating PI3K/AKT pathway	[98, 124]
Nasopharyngeal carcinoma	Human/patient tissues and cell line	HDAC7 was upregulated in cancer tissues and correlated with progression and poor prognosis	HDAC7 promotes tumour cell proliferation, migration and invasion by inhibiting miR‐4655	[125]
	Mouse/xenograft model	*Hdac7* knockdown reduced tumour growth	N/A	
Breast cancer	Human/patient tissues and cell line	*HDAC7* level was elevated in cancerous tissues, protein level was increased in recurrent breast cancer serum	N/A	[127, 128]
		*HDAC7* was upregulated in cancer stem cells, promoted chemotherapy resistance and inhibited autophagy	HDAC7 and HDAC1 deacetylate HSP70	[127]
		HDAC7 sustained cancer cell proliferation and repressed cytokines in tumour microenvironment	HDAC7 inhibition reduces H3K27ac and expression of super enhancer‐associated oncogenes in cancer stem‐like cells	[101, 131]
Blood cancer	Human/patients bone marrow, leukaemic cell line	Dysregulated HDAC7 was associated with lymphoblastic leukaemia and was correlated with poor prognosis	HDAC7 represses oncogenic activity of C‐MYC and induces apoptosis	[46, 134, 135, 136, 137]
	Mouse/xenograft model	Hdac7 inhibited tumour growth	N/A	[46]
Autoimmune, inflammatory and metabolic diseases				
Autoimmune disease				
Systemic lupus erythematosus	Human/patient genome analysis	Variation in *HDAC7* was related to disease pathogenesis	SNP was identified by whole exome sequencing	[139]
Inflammatory bowel disease	Mouse/ B6, BoyJ, Vα14/Jα18, Hdac7‐ΔP and PLZF transgenic mice, *Hdac7^flox:−^::lck^cre^*	Blocking Hdac7 nuclear export caused lethal autoimmune phenotypes	Nuclear Hdac7 suppresses MAPK pathway activation upon TCR activation. Hdac7 interacts with PLZF and suppresses its transcriptional activity in iNKT	[66, 72]
Inflammatory and fibrotic diseases				
Osteoarthritis	Human/patient tissues and cell line	HDAC7 was elevated in patients' cartilage	HDAC7 promotes expression of MMP‐13, contributing to cartilage degradation	[140]
Systemic sclerosis	Human/patient B cells and primary cell	*HDAC7* was decreased in B cells from SSc patients. siRNA silencing *HDAC7* suppressed collagen production in fibroblast	HDAC7 suppresses type I and III collagen gene transcription and expression	[142, 143]
Peyronie's disease	Human/patient tissues and primary cell	*HDAC7* was higher in PD patients. Inhibiting HDAC7 reduced fibrotic extracellular matrix production and fibroblast differentiation	HDAC7 may promote transcriptional activation in TGF‐β1 fibrotic pathway	[144]
Diabetes				
Type 2 diabetes	Human/patient tissues	*HDAC7* was increased in pancreatic cells and was negatively correlated with insulin secretion	N/A	[146]
	Mouse and rat/primary cell	*Hdac7* silencing reduced liver glucogenesis and dysregulated insulin secretion in pancreatic cells	Hdac7 induces glucogenesis, its overexpression enhances β‐cell apoptosis and reduces insulin production	[28, 146]
Liver disease				
Hepatic steatosis	Human/cell line	Recruitment of HDAC7 to *NUR77* promoter was decreased in homocysteine treated hepatic cells	HDAC7 deacetylates H3K27 in the promoter of *NUR77*	[148]
Liver fibrosis	Human/primary cell	CLYD restrained HDAC7 in cytoplasm and induced HGF expression. HGF attenuates liver fibrosis	HDAC7 binds to *HGF* promoter and repressed *HGF* transcription	[150]
	Mouse/*CYLD^−/−^* mice and primary cell	*CYLD^−/−^* mice had lower HGF level and were susceptible to liver inflammation, injury and fibrosis		
Primary sclerosing cholangitis	Human/patient genome analysis	SNP in *HDAC7* gene was associated with disease	N/A	[151]
Respiratory disease				
Cystic fibrosis	Human/cell line	Silencing *HDAC7* restored channel activity of deficient ∆F508‐CFTR	HDAC7 might transcriptionally regulate signalling pathways related to CFTR folding, maturation, trafficking and channel activity	[158]
Acute lung injury	Mouse/infection model	*Hdac7* silencing ameliorated inflammation in bacterial infection‐induced acute lung injury	N/A	[159]
Neurological conditions				
Huntington's disease	Mouse and rat/R6/2 mice and mouse/rat primary cell	Overexpressing Hdac7 blocked neuronal cell death	Hdac7 binds to the *c‐Jun* promoter and inhibits *c‐Jun* transcription, leading to suppressed apoptosis	[165]

### HDAC7 in cancer

Aberrant expression and/or function of HDAC7 has been linked to cancer, including colorectal, gastric, pancreatic and brain cancer. The dysregulation of HDAC7 in pancreatic adenocarcinomas, gastric cancers and colorectal cancer makes it a potential biomarker for progression of these cancers [96, 97, 98, 99]. Some evidence suggests that HDAC7 may have causal roles in driving tumour growth and/or progression, for example by promoting oncogenic signalling [100] and/or driving chronic inflammatory responses [101].

#### Colorectal cancer

Multiple studies have linked HDAC7 to colorectal cancer (CRC), which results from the accumulation of both genetic and epigenetic aberrations [102, 103]. Two studies examining resected colon tissues found that *HDAC7* gene expression was significantly increased in rectal biopsies from patients with colorectal adenomas versus those without dysplasia [96], and in biopsies of patients with CRC versus healthy colon mucosa [97]. This suggests that *HDAC7* may be a potential diagnostic and/or prognostic marker for CRC. Further, another study of resected patient CRC tissues reported that the microRNA, miR‐489, which represses *HDAC7* expression, was significantly downregulated in tumour tissues by comparison to adjacent noncancerous tissues [104]. The capacity of miR‐489 to repress HDAC7 mRNA and protein expression was verified experimentally in HEK293 cells, with overexpression of miR‐489 reducing HDAC7 mRNA and protein levels. Importantly, HDAC7 promoted tumour cell proliferation and invasion in both CRC cell lines and mice, implying that it may have a functional role in CRC progression.

#### Pancreatic cancer

Pancreatic cancers are categorised according to their malignancy and the lesion where pathogenesis occurs. *HDAC7* mRNA levels in tumour tissues from patients with pancreatic adenocarcinomas (PA), the most common type of pancreatic cancer, were significantly elevated compared with normal/benign pancreatic tissues or chronic pancreatitis patient tissues [99, 105]. HDAC7 protein levels were similarly elevated in PA tissues. Furthermore, shRNA‐mediated downregulation of HDAC7 reduced cell growth of a human pancreatic tumour cell line [105]. Interestingly, mRNA levels of *NUR77*, which is known to be regulated by HDAC7 in other cellular contexts [45], were also increased in PA tissues [105]. However, precise mechanisms by which HDAC7 may contribute to PA progression remain unclear.

#### Liver cancer

Genetic analyses have also revealed an association between *HDAC7* and hepatocellular carcinoma (HCC) [106]. *HDAC7* mRNA levels were significantly increased in several human and murine HCC cell lines compared to hepatocytes. By analysing two human datasets, the authors found that *HDAC7* levels were also elevated in tumours from HCC patients by comparison to non‐tumour tissues. Moreover, elevated *HDAC7* expression correlated with poor patient outcomes. One possible mechanism may involve regulation of collagen production, as their analysis identified a strong correlation between expression of *HDAC7* and that of collagen alpha 1, an extracellular matrix protein associated with fibrosis [107]. Finally, gene expression analysis of tumorous livers from *Trim24* ^−/−^ mice, which spontaneously progress from steatosis to HCC [108], revealed that *Hdac7* expression was elevated compared to non‐tumorous tissues. These observations, along with others linking HDAC7 to liver fibrosis (see below), suggest that hepatic HDAC7 may have a pathogenic role in the development of chronic liver diseases and its progression to liver cancer.

#### Glioblastoma multiforme

Glioblastoma multiforme (GBM) is the most aggressive and common primary astrocytoma, accounting for more than 60% of adult brain tumours [109, 110]. Genetic [111] and epigenetic abnormalities [112] are intimately linked to pathogenesis, with an imbalance between histone acetylation and deacetylation being thought to contribute to glioma oncogenesis [112]. *HDAC7* mRNA expression was significantly elevated in human GBM compared to normal brain tissues [113]. This increased expression, along with that of *STAT3*, was also associated with poor survival for a subgroup of patients [114]. Despite an apparent association between *STAT3* and *HDAC7* expression, mechanistic studies revealed that silencing HDAC7 in GBM cell lines increased STAT3 protein levels, as well as its phosphorylation. This resulted in a STAT3‐mediated anti‐angiogenic phenotype and a reduction in tumour growth *in vivo*. Thus, HDAC7 may promote tumour growth via sustaining angiogenesis. Another potential mechanism may involve promoting glioma cell proliferation and invasion. In 81 of 133 patient glioma samples examined, nuclear expression of zinc‐finger protein‐326 (ZNF326), which promotes cancer cell growth [115], was elevated and positively correlated with tumour malignancy [100]. Increased ZNF326 upregulated the expression of HDAC7, allowing it to deacetylate β‐catenin, resulting in the nuclear localisation of this transcription factor. Of note, β‐catenin is a central signal transducer in the oncogenic Wnt signalling pathway that is associated with many cancers [116], including glioblastoma [117, 118, 119]. Thus, HDAC7 might also promote GBM by inducing Wnt‐dependent oncogenic genes such as *AXIN2*, *MYC* and *MMP7*.

#### Lung cancer

Lung cancer is the leading cause of cancer death worldwide [120]. It is mainly categorised into two subtypes, small cell lung cancer and non‐small cell lung cancer (NSCLC), with NSCLC accounting for the majority of lung cancers [121]. Lei et al. [35] reported a positive correlation between elevated HDAC7 mRNA and protein expression and poor prognosis in lung cancer, through their analysis of 484 lung cancer patient samples. Using both a mouse model of NSCLC and *in vitro* experiments, their study provided evidence that HDAC7 may also have a role in lung tumorigenesis. The number of lung tumours in heterozygous *Hdac7* ^+/−^ */K‐Ras* mice was significantly reduced compared to *K‐Ras* control mice. Consistent with this, lung tumour cell proliferation was reduced, while tumour cell apoptosis was enhanced. Similar phenotypes were also observed in human lung cancer cell lines. Mechanistically, HDAC7 inhibited the tumour‐suppressive activity of STAT3, similar to the aforementioned findings in GBM [114]. In this context, HDAC7 appears to contribute to lung tumorigenesis by directly deacetylating STAT3 and inhibiting its phosphorylation. On the other hand, a subsequent study revealed that HDAC7 can also promote NSCLC through maintaining the cytoskeletal structure and angiogenesis during neovascularisation of endothelial progenitor cells [122]. Thus, HDAC7 likely supports the development and progression of lung cancer by multiple mechanisms in various cell types.

#### Gastric cancer

Gastric cancer (GC) is the fourth most common malignant cancer [123]. HDAC7 protein levels in gastric adenocarcinoma tissues were significantly reduced compared to the adjacent non‐cancerous tissues. Interestingly however, high HDAC7 levels in cancerous tissues correlated with distant metastasis and poorer prognosis [98]. Recently, another study using tissues from GC patients reported increased HDAC7 mRNA and protein expression compared to surrounding normal tissue from the same patients, and high HDAC7 levels were again associated with poor patient survival [124]. Hence, both of these studies identified a correlation between HDAC7 expression and GC progression. Furthermore, silencing or overexpressing HDAC7 in human gastric carcinoma AGS cells impeded or promoted cell proliferation and invasion, respectively [98]. At a molecular level, the tumour suppressor miR‐489 was dramatically reduced in cancerous biopsies and cell lines, implying potential overlap with the mechanisms implicated in CRC development (see above). Reduced miR‐489 led to derepression of *HDAC7* expression and subsequent activation of the PI3K/AKT pathway, a signalling pathway with a well‐known role in tumour development [124].

#### Nasopharyngeal carcinoma

A recent study comparing nasopharyngeal carcinoma tissues with normal nasopharyngeal mucosa tissues revealed that HDAC7 was correlated with disease progression and poor prognosis [125]. Overexpressing HDAC7 in the cancer cell lines HK1 and 5–8F enhanced cell proliferation, migration and invasion. Further, *HDAC7* knockdown decreased *in vivo* tumour growth in a mouse xenograft model. Distinct from its regulation by miRNAs in colorectal and gastric cancers (see above), HDAC7 acted as an upstream regulator of miR‐4465 to promote this type of cancer. This microRNA suppresses EphA2, a well‐defined tumour promoter [126]. It was shown that HDAC7‐mediated inhibition of miR‐4465 increased EphA2 expression in HK1 and 5‐8F cells, providing a plausible mechanism for the pro‐tumorigenic effects of HDAC7.

#### Breast cancer

Abnormal expression of HDAC7 has also been demonstrated in breast cancer. *HDAC7* mRNA was elevated in breast cancer tissues by comparison with normal breast tissue [127]. Another study compared serum samples from patients with recurring versus nonrecurring breast cancer, finding that protein levels of HDAC7 were significantly higher in patients with recurring disease [128]. Similarly, HDAC7 protein levels were increased in cancer stem cells (CSCs) versus non‐stem tumour cells [129]. CSCs remain a challenge for effective treatment of breast cancer due to their capacity to regenerate non‐stem cancer cells, resist chemotherapies and radiotherapies, and contribute to cancer recurrence [130]. HDAC7 is thought to promote breast cancer cell survival through its capacity, in cooperation with HDAC1, to deacetylate HSP70, which limited autophagic cell death of CSCs [127]. Increased HDAC7 levels also helped maintain the stem cell phenotypes of sphere formation and cell proliferation. This is likely a result of upregulating stem cell transcription factor‐encoding genes and super‐enhancer associated oncogenes [101, 131]. In non‐stem cells, HDAC7 supported a microenvironment for mammary epithelial cells to sustain their proliferation, invasion and stemness during the transformation process. In this regard, HDAC7 repressed the transcription of a range of chemokines and cytokines, for example the anticancer cytokine IL‐24 [101] that promotes tumour cell killing [132]. Other evidence also suggests that HDAC7 may contribute to breast cancer progression through immune modulation. Guerriero et al. [133] showed that a class IIa HDAC inhibitor enhanced macrophage‐mediated anti‐tumour responses in a mouse breast cancer model, suggesting that HDAC7 and/or other class IIa HDACs may dampen tumoricidal activity of macrophages. Collectively, these studies reveal multifaceted roles for HDAC7 in breast cancer. HDAC7 is positively correlated with breast cancer recurrence, it promotes the transformation of epithelial cells, it appears to contribute to the proliferation and maintenance of CSC stemness, and it may limit anti‐tumour responses of immune cells. Interestingly, HDAC7 also maintained the CSC phenotype in ovarian cancer cell lines [129].

#### Blood cancers

Given the crucial role of HDAC7 in lymphoid development (see above), it is not surprising that dysregulation of HDAC7 expression is linked to several blood cancers, particularly B lineage‐derived malignancies. Low levels of HDAC7 expression were reported in the bone marrow of patients with pro‐B acute lymphoblastic leukaemia compared to those from healthy controls, as well as in various malignant B cell lines [46]. *In vitro* and *in vivo* studies have shown that HDAC7 has an anti‐oncogenic function in B‐malignant cell lines and in a xenograft model. Mechanistically, HDAC7 induced apoptosis‐associated genes, which was likely a result of repressing the oncogene *C‐MYC*. Interestingly, *HDAC7* was overexpressed in patients with both acute [134] and chronic [135, 136] lymphoblastic leukaemia, and its expression correlated with a poorer prognosis in both young and adult patients. A more recent study profiling gene expression in patients with acute promyelocytic leukaemia found that *HDAC7* was co‐expressed with the tumour‐associated gene *SHB*, again correlating with poor survival [137]. These clinical studies suggest that HDAC7 likely has multiple roles in different types of blood malignancies. HDAC7 has also been pursued as a target in chronic lymphocytic leukaemia through reprogramming of macrophage functions to boost anti‐tumour responses [138].

### HDAC7 in autoimmune, inflammatory and metabolic diseases

Genetic studies have linked abnormal *HDAC7* expression or variation with multiple autoimmune and inflammatory diseases. Investigations into underlying mechanisms are ongoing, however, likely mechanisms include effects on signalling, gene regulation and cell metabolism in both immune and non‐immune cells.

#### Autoimmune diseases

Whole exome sequencing of six index patients with early‐onset or familial systemic lupus erythematosus revealed a correlation with homozygous missense variants in the coding regions of six proteins, among which the variant in *HDAC7* (c.238C>T) had a strong correlation with disease severity [139]. A child carrying a homozygous mutation (p.Arg80Cys) in a highly conserved region of *HDAC7* (chr12:48192588G>A) was the only symptomatic patient in the family. The parents were heterozygous and the siblings were either heterozygous or negative for this variant. It was therefore hypothesised that this genetic alteration in *HDAC7* predisposes to autoimmunity; however, a causative link remains to be established.

More recently, a mechanistic study in mice revealed that perturbations in Hdac7 function can contribute to autoimmunity and a phenotype resembling inflammatory bowel disease (IBD) by altering CD4/CD8 DP thymocyte and iNKT cell development. As described above, HDAC7 is exported from the nucleus upon TCR activation. Transgenic mice expressing a mutant of Hdac7 that was retained in the nuclear compartment resulted in a lethal autoimmune phenotype, characterised by excessive pathology in the liver, exocrine pancreas and digestive organs [66]. This mutation allowed T cells to escape from negative selection and to attack healthy organs. Interestingly, mice carrying the same mutation failed to generate iNKT in the liver, digestive system and pancreas. This lack of iNKTs further increased the vulnerability of these organs to autoimmune attack. These findings thus reveal that nuclear export of Hdac7 is critical for normal iNKT development and homeostasis [72]. Perturbations in this pathway may thus contribute to autoimmune conditions.

#### Inflammatory and fibrotic diseases

Increasing evidence implicates HDAC7 in several other inflammation‐related diseases. Levels of HDAC7 were elevated in cartilage samples from ten osteoarthritis (OA) patients compared to six healthy donors [140]. Silencing of HDAC7 in a human chondrosarcoma cell line reduced the expression of matrix metalloproteinase‐13, a protease that degrades collagen and contributes to OA pathogenesis. This study therefore implicates HDAC7 as a promoter of collagen degradation during OA. Systemic sclerosis (SSc) and Peyronie's disease (PD) are inflammatory diseases caused by excessive fibrosis in the skin or internal organs. SSc is an autoimmune disease in which B lymphocytes contribute to pathogenesis by secreting cytokines and activating other immune cells [141]. Genetic analysis of B cells from SSc patients found that histone H4 was hyperacetylated and *HDAC7* was significantly decreased compared to healthy donors [142]. However, the functional significance of this is unclear. In contrast, although HDAC7 mRNA and protein levels were not dysregulated in SSc skin fibroblasts, siRNA‐mediated silencing of *HDAC7* in cultured SSc fibroblasts reduced both constitutive and transforming growth factor beta‐1 (TGF‐β1)‐inducible production of type I and III collagens [143]. *HDAC7* mRNA levels were also found to be elevated in fibroblasts isolated from plaques of PD patients. Silencing of *HDAC7* in PD fibroblasts limited the fibroblast to myofibroblast transition and inhibited the production of fibrotic extracellular matrix proteins including fibronectin, PAI‐1, collagen I and IV. In this regard, HDAC7 may drive fibrotic progression by inducing the nuclear accumulation of Smad proteins, which are key signal transducers in TGF‐β1‐inducible fibrotic pathways [144]. Therefore, HDAC7 may promote dermal fibrosis by inducing collagen production and via other uncharacterised cell type‐specific mechanisms.

#### Diabetes

In addition to its role in dysregulated inflammatory responses, HDAC7 plays an important role in metabolic homeostasis. Dysregulated glucose metabolism is linked to many conditions, for example type 2 diabetes (T2D), which is characterised as a low‐grade inflammatory and metabolic disorder [145]. Hdac7, as well as two other class IIa HDACs (Hdac4 and Hdac5), induced glucogenesis in the liver in response to glucagon signals [28]. Mice depleted of Hdac4/5/7 in their livers had reduced blood glucose, elevated liver glycogen and improved glucose tolerance. In a mouse model of T2D (*db/db* and *ob/ob*), simultaneous silencing of these three HDACs reduced fasting blood glucose and improved glucose tolerance, suggesting that Hdac7, together with Hdac4 and Hdac5, may be crucial in regulating glucose homeostasis.

Another mechanism by which HDAC7 can regulate metabolism is via control of insulin secretion. *HDAC7* expression in pancreatic islets from T2D patients was elevated by comparison with non‐diabetic donors, and high protein levels negatively correlated with insulin secretion by human islets [146]. *In vitro* experiments also confirmed that overexpressing Hdac7 in both rat islets and clonal β‐cells impaired insulin secretion. Higher levels of Hdac7 in clonal β‐cells perturbed mitochondrial functions, but enhanced the apoptosis of β‐cells, both of which contribute to T2D. Consistent with a role for HDAC7 in metabolic dysfunction, the reported class IIa HDAC inhibitor MC1568, rescued glucose‐induced insulin production in T2D patient islets [147]. Such data suggest that targeting HDAC7 could be one approach to correct insulin production and secretion in T2D.

#### Liver diseases

HDAC7 has an important role in the liver as an epigenetic modifier and/or non‐deacetylase, at least during development of this organ. Correlations between HDAC7 and the progression of chronic liver disease have been widely studied, from simple steatosis to hepatic cell activation to cancer. In an *in vitro* model of hepatic steatosis, treatment of HepG2 cells with homocysteine rapidly and transiently decreased the recruitment of HDAC7 to the *NUR77* promoter. This led to H3K27 hyperacetylation at the *NUR77* promoter and increased *NUR77* expression, which was associated with a reduction in lipid accumulation and steatosis. Therefore, HDAC7 may contribute to hepatic steatosis by inhibiting *NUR77* expression, thus promoting lipid accumulation in hepatocytes [148]. Steatosis in the liver is resolvable if risk factors are removed; however, repeated liver injury can result in a transition from steatosis to fibrosis, which can eventually lead to HCC [149]. Liver inflammation and hepatic stellate cell activation are central to these processes. In activated hepatic stellate cells, HDAC7 localised to the cytoplasm through an interaction with the tumour‐suppressor cylindromatosis (CYLD), thus derepressing hepatocyte growth factor (HGF) expression [150]. HGF protects from liver injury‐mediated fibrotic progression [107]. The capacity of CYLD to antagonise HDAC7‐mediated repression of HGF expression limited hepatic fibrosis, suggesting a potential role for HDAC7 as a switch that enables gene expression upon liver injury. In fact, an intronic SNP within the *HDAC7* gene (rs11168249) at chromosome 12q13 was strongly associated with primary sclerosing cholangitis [151], a severe liver disease characterised by inflammation and fibrosis. Interestingly, this GWAS study also identified two disease‐associated SNPs within the intron of *PKD2* (rs60652743 at 19q13) and *SIK2* (rs7937682 at 11q23). PKD2 and SIK2 have both been reported as upstream regulators of HDAC7. Pkd2 phosphorylated Hdac7, which led to *Nur77* derepression and negative selection of TCR‐activated mouse thymocytes [49], whereas the SIK2 serine/threonine kinase can phosphorylate class IIa HDACs, leading to MEF2‐dependent transcription [152] and glucose uptake by adipocytes [153]. Thus, it is possible that these three loci are mechanistically connected and that HDAC7 may contribute to hepatic inflammation via Nur77/MEF2‐mediated inflammation and metabolic signalling pathways. Interestingly, the same SNP within *HDAC7* was also associated with IBD [154].

### HDAC7 in respiratory diseases

In the lung, HDAC7 was reported to be a potential target for treating cystic fibrosis. This genetic disease is caused by mutations in the gene encoding cystic fibrosis transmembrane conductance regulator (CFTR). Five classes of more than 2000 mutations in *CFTR* have been associated with various phenotypes and disease severity in cystic fibrosis [155]. Deletion of F508 (∆F508) in one or both *CFTR* alleles causes misfolding of CFTR, thereby impairing its biological function as a chloride channel at the cell surface [156, 157]. siRNA‐mediated silencing of HDAC7 in a bronchial epithelial cell line increased the stability and trafficking of the ∆F508‐CFTR protein and restored its activity as a chloride channel, although the exact mechanism by which this occurs is unknown [158]. Gene expression analysis of these cells revealed transcriptional alterations in signalling pathways related to protein folding, maturation, trafficking and channel activation of CFTR, thus suggesting that HDAC7 silencing rescues cell surface ∆F508‐CFTR expression by enhancing correct folding and stabilisation of CFTR and/or promoting clearance of the misfolded ∆F508. A recent study also reported a role for HDAC7 during lung infection. Silencing of *Hdac7 in vivo* ameliorated inflammatory responses and improved survival in an *Escherichia coli*‐induced acute lung injury model, suggesting that Hdac7 drives systemic inflammation during infection [159].

### Role of HDAC7 in neurological conditions

Unlike many of the conditions described above, HDAC7 may have a protective rather than detrimental role in diseases of the central nervous system. Studies with HDAC inhibitors have implicated HDACs in neuron function and neurological disease. Broad‐spectrum HDAC inhibitors (trichostatin A, sodium butyrate) increased expression of apoptotic genes in neuronal cells [160], and therefore induced stress responses in rat cerebellar granule neurons and mouse neuroblastoma cells cerebellar granule neurons [161], leading to cell death. This suggests that one or more HDACs may protect against neuronal cell death. On the other hand, HDAC inhibitors were protective in mouse models of polyglutamine disorders, such as Huntington's disease (HD) [162]. Administration of the broad‐spectrum HDAC inhibitor suberoylanilide hydroxamic acid (Vorinostat) to R6/2 transgenic mice, which express exon 1 of the human HD gene and exhibit human HD‐like phenotypes, increased protein acetylation in the brain and rescued motor damage [163]. This indicates that one or more HDACs may contribute to pathology in this model. Vorinostat treatment also reduced *Hdac7* expression in the brains of R6/2 mice, suggesting that this may contribute to the efficacy of this HDAC inhibitor. However, knock‐out of one *Hdac7* allele on the R6/2 background did not improve physiological or behavioural HD‐associated phenotypes [164]. Of note, Ma and D'Mello [165] reported that apoptotic stimuli decreased Hdac7 protein levels in cultured cerebellar granule neurons and that ectopic expression of Hdac7 in these cells blocked apoptosis. Mechanistically, Hdac7 supports neuronal survival by directly binding to the promoter of *c‐Jun* to inhibit its transcription, consistent with a known functional role for *c‐Jun* in neuronal cell apoptosis [166]. The capacity of Hdac7 to support neuron survival through repression of *c‐Jun* occurs via a deacetylase‐independent mechanism. Collectively, these findings suggest that HDAC7 may have a protective role in limiting neurodegeneration. Given that c‐Jun functions in many cell types, it may also be of interest to examine Hdac7‐mediated control of this transcription factor in other biological contexts, for example during T cell and muscle differentiation.

## HDAC7 inhibitors

The general status of HDAC inhibitors [25] and the particular challenges in inhibiting class IIa HDACs [20, 167] have been described previously. The specific effects of inhibitors of class IIa HDACs in physiology and disease have been recently reviewed elsewhere [20], so instead, we summarise here some of the most potent inhibitors of HDAC7 reported to date.

Crystal structures have been reported for human HDAC7 (PDB codes: 3C0Y, 3C0Z, 3C10 [22]; 3ZNR, 3ZNS [168]) and HDAC4 (PDB codes: 5ZOO, 5ZOP [169]; 4CBT, 4CBY [170]; 6FYZ [171]; 5A2S [172]) and they reveal key differences from class I HDACs. All class IIa HDACs lack the acetate release channel of class I HDACs that promotes efficient product dissociation and rapid substrate turnover, and class IIa HDACs tend to have only ~ 0.1% of the catalytic activity of class I HDACs. This low activity has necessitated the use of an artificially activated substrate (e.g. trifluoroacetylated lysine analogues) to measure the activation and inhibition of HDAC7 and other class IIa HDACs in cell‐free media. Using such an assay, a few potent inhibitors of HDAC7 enzymatic activity have been identified (exemplified by inhibitors shown in Table 4), but they also inhibit other class IIa HDACs (HDAC4, 5, 9) and even show some inhibition of class I HDACs. These inhibitors all exploit a key structural difference in the catalytic site of class IIa HDACs, where there is a His in place of a Tyr that is present in class I HDACs [24]. This causes a conformational change that creates an additional cavity adjacent to the catalytic zinc. All potent HDAC7 inhibitors exploit the larger active site, often by presenting a substituent that occupies this additional pocket. The majority of HDAC7 inhibitors use a hydroxamic acid to bind to zinc, but an unusual trifluoromethyloxadiazole group (TFMO) also has been used to effectively coordinate to zinc.

**Table 4:** Structures and reported potencies (IC_50_, nm) for inhibitors of human HDAC7 and other human HDAC isozymes (class IIa selectivity‐conferring group in blue).

HDAC		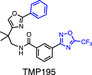	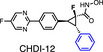	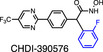	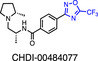	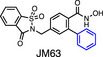
I	1	> 100 000	14 000	39 700	17 000	4900
	2	> 100 000	> 50 000	> 50 000	27 000	–
	3	> 100 000	7400	25 800	10 000	–
	8	11 700	280	9100	2000	71
IIa	4	111	10	54	10	49
	5	106	10	60	20	8
	7	46	30	31	20	12
	9	9	60	50	30	38
IIb	6	47 800	3100	6200	22 000	5300
	10	> 100 000	–	–	–	–
III	11	> 100 000	–	–	–	–
Reference		[168]	[172]	[171]	[176]	[177]

A high‐throughput screen of 2 million compounds by Tempero Pharmaceuticals identified compounds with a TFMO motif, exemplified here by TMP195 (Table 4), which are potent inhibitors of HDAC7 and other class IIa HDACs [168]. A crystal structure of an analogue bound to HDAC7 (PDB code: 3ZNR) showed that the bulky TFMO component (marked in blue) was bound to the zinc within the active site, and the ligand adopted an unusual U‐shaped conformation that caused the terminal phenyl ring (blue) to occupy the unique class IIa cavity [168]. TMP195 altered the secretion of the chemokines CCL1 and CCL2 from human monocyte‐derived macrophages and regulated the expression of far fewer genes than Vorinostat in lymphocytes, suggestive of selective inhibition of class IIa HDACs in cells. As noted earlier, it also reduced breast tumours and metastases by recruiting and differentiating antitumour macrophages in mice [133].

Researchers at the Cure Huntington's Disease Initiative (CHDI) Foundation have reported three series of potent brain‐penetrating inhibitors of HDAC4. Exemplified by CHDI: 12, 390576, 00484077 (Table 4), they also potently inhibited HDAC7. The two hydroxamates feature a central cyclopropyl (e.g. CHDI‐12) [170, 172] or methine (e.g. CHDI‐390576) [171] scaffold that each project a phenyl ring (marked blue in Table 4) into the unique selectivity‐determining pocket of class IIa HDAC enzymes, as shown in crystal structures bound to HDAC4 (PDB codes: 4CBT, 4CBY [170]; 6FYZ [171]; 5A2S [172]). CHDI‐390576 exhibited improved selectivity over the unusually malleable [173] class I enzyme HDAC8, which lies at the phylogenetic border of class I and II enzymes [174]. Unlike other class I HDACs (HDAC 1–3), HDAC8 can accommodate bulkier substituents near the zinc binding group [175]. In 2021, CHDI reported a series of TFMO‐based compounds, with CHDI‐00484077 exhibiting 10‐ and 2‐fold improved inhibition of HDAC4 and HDAC7 over TMP195, respectively [176]. It maintained excellent selectivity for class IIa HDACs despite being less likely to adopt the U‐shaped conformation observed with TMP195. Although the unbound fraction in mouse brain was much higher for the TFMO compound than the corresponding hydroxamate (0.17 vs < 0.026), the three CHDI compounds in Table 4 exhibited moderate‐to‐high oral bioavailability in mice (44–100%), comparable durations (12–14 h at 10 mg·kg^−1^; 100 mg·kg^−1^ for TFMO compound) for which the brain concentration was higher than IC_50_ values for class IIa HDACs in cells. Therefore, these compounds may be potentially useful for studying HDAC7 functions in brain.

JM63 is member of a series of benzoyl hydroxamate inhibitors that are the most potent HDAC7 inhibitors reported to date [177]. Like CHDI‐12 and CHDI‐390576, a protruding phenyl substituent adjacent to the Zn^2+^‐binding hydroxamate conferred selectivity for class IIa over class I HDACs, as measured by human enzyme inhibition and human cell‐based hyperacetylation assays, albeit with modest selectivity over HDAC8. JM63 was a 10‐fold more potent inhibitor of class IIa HDACs than TMP195 in cells and possessed greater stability in cytochrome P450‐rich rat liver microsomes (*t*_1/2_ 230 vs 46 min). Consistent with a pro‐inflammatory role of HDAC7 in human monocyte‐derived macrophages [29], JM63 attenuated LPS‐induced secretion of inflammatory cytokines, although the HDAC isoform(s) responsible for inflammatory mediator production were not reported.

**Fig. 4:**
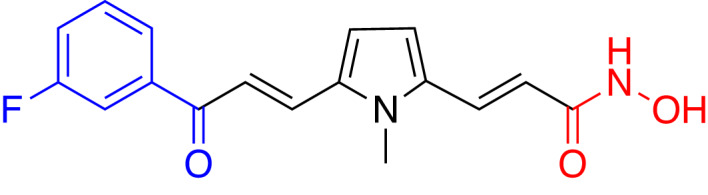
Reassigned structure of MC1568. Correctly assigned regiochemistry for linker (black) that separates capping group (blue) from zinc‐binding hydroxamate group (red).

MC1568 is a hydroxamate‐based HDAC inhibitor sold commercially as a selective inhibitor of class IIa HDACs, including HDAC7 [147]. After its initial report [178], its structure was reassigned (Fig. 4) [179], but it possesses no component that can be considered to confer selectivity for class IIa HDACs. It is unlikely to selectively inhibit class IIa HDACs. Some studies report that it inhibits human HDAC4 (at 5 μm) [178, 180], but later information indicated inhibition (up to 10 μm) of HDAC6 but not HDAC4 (nor HDAC 3) [179]. Another study found that MC1568 (up to 10 μm) did not inhibit any of the four class IIa HDACs, but instead inhibited HDAC8 (IC_50_ 151 nm) [181]. We do not advise the use of MC1568 for investigating class IIa HDACs *in vitro* or *in vivo*.

To date, HDAC7 inhibitors have mainly been studied in cancer, with emerging reports on regulatory effects in the central nervous system and neurodegenerative conditions, and potential roles in inflammatory conditions and metabolic dysfunction [20]. Clearly, based on the information collected earlier in this review for the importance of HDAC7, there are many new possible applications for an effective HDAC7 inhibitor.

## Final perspectives

Dysregulated HDAC7 expression and/or genetic variation in the *HDAC7* locus has been correlated with the severity and progression of many diseases, making it a potential biomarker and/or target. Five HDAC inhibitors (Vorinostat, Belinostat, Romidepsin, Tucidinostat and Panobinostat) have been approved to treat both lymphomas and myelomas, with more pan‐ and selective inhibitors under clinical trials for various cancers and non‐oncogenic diseases, including inflammatory disease, neurodegenerative disease and muscular dystrophy [182]. Hence, it may be feasible to selectively target HDAC7 for cancer and/or other diseases in which this class IIa HDAC has been implicated. But how far are we from achieving this goal and what are the obstacles?

Firstly, we need to have a precise understanding of the molecular mechanisms by which HDAC7 functions in different disease contexts. At a mechanistic level, HDAC7 alters gene transcription by modification of both histone and non‐histone targets. Repression, derepression or activation of genes by HDAC7 contributes to a range of physiological and pathological processes related to cell differentiation, proliferation, activation and/or death. HDAC7 modifies specific substrates through its deacetylase and/or SUMOylation‐promoting activities, as well as by acting as a scaffolding protein. Some of these effects lead to cross‐talk with other post‐translational modifications, as exemplified by altered phosphorylation of STAT3 resulting in inhibition of gene expression [35, 114]. Given these wide‐ranging biological activities, it is important to understand specific molecular mechanisms by which HDAC7 contributes to different disease states. In many cases, this goal remains elusive. Most progress has been made in understanding how HDAC7 influences endothelial cell, muscle and lymphocyte functions. More recent evidence implicates HDAC7 in myeloid cell biology, particularly in metabolic and inflammatory responses [26, 29].

Cellular functions of HDAC7 are highly dependent on its subcellular localisation and environmental cues. Consequently, its roles in different cell types can diverge dramatically. Many outstanding questions relating to HDAC7 biology remain. For example, there are major knowledge gaps about which specific phenotypes associated with treatment with broad‐spectrum HDAC inhibitors are phenocopied by more selective pharmacological targeting of HDAC7. It will also be of interest to document overlapping biological effects of class IIa HDAC inhibitors and genetic targeting of *HDAC7*. Furthermore, in many cases, it is unclear if phenotypes caused by HDAC7 manipulation are associated with its deacetylase and/or scaffolding functions. It is also important to consider compensatory roles for other class IIa HDACs members in specific biological responses [23]. Another challenge in investigating HDAC7 biology relates to its essential role in development [3], given that global *Hdac7* knockout causes embryonic lethality [43]. Hence, a combination of ‐omics analyses, tissue‐specific knock‐out studies, *in vivo* approaches using HDAC7‐selective inhibitors and detailed mechanistic *in vitro* studies will be required to understand how this multifunctional protein controls so many distinct physiological and pathophysiological processes. Continued elucidation of mechanisms by which HDAC7 contributes to dysregulated homeostasis will undoubtedly deliver further molecular insights into a range of cancers, inflammatory diseases and metabolic disorders and may ultimately validate HDAC7 as a target in one or more of these conditions.

## Conflict of interest

The authors declare no conflict of interest.

## Author contributions

YW, RA, JYWM, KDG, DR, DK, DPF and MJS all contributed to the writing and editing of this manuscript.

## Data Availability

Data sharing not applicable to this article as no datasets were generated or analysed during the current study.
